# Effectiveness of WeChat Public Account Intervention Based on the Information-Motivation-Behavioral Skills Model Among College Students With Internet Addiction: Randomized Controlled Trial

**DOI:** 10.2196/84664

**Published:** 2026-07-03

**Authors:** Huayu Yang, Anyi Geng, Wenhua Ruan, Yuhan Yang, Fangzheng Xu, Haiyan Shi, Wenzhuo Xu, Kele Jiang, Hao Guo, Sainan Wang, Zheng Hu, Mengting Man, Zhihua Zhang

**Affiliations:** 1 Department of Epidemiology and Biostatistics School of Public Health Anhui Medical University Hefei, Anhui China; 2 Center for Disease Control and Prevention of Liuhe District Nanjing, Jiangsu China; 3 Department of Public Health and Health Administration Clinical College of Anhui Medical University Hefei, Anhui China

**Keywords:** information, motivation, behavioral skills model, IMB model, internet addiction, intervention, randomized controlled trial, WeChat public account

## Abstract

**Background:**

Internet addiction (IA) among college students causes multiple harms and increases suicide risk. Effective interventions can reduce these and have public health value.

**Objective:**

This study evaluates a WeChat public-account intervention based on the information, motivation, behavioral skills (IMB) model to reduce IA in college students and to examine mechanisms driving behavior change.

**Methods:**

A total of 226 college students aged 18 to 24 years with IA were recruited from a university in Anhui Province, China, and randomized (stratified by gender) to intervention (n=113) or control (n=113) groups. The intervention comprised a 6-week structured online program delivered via a WeChat public account, organized into 3 modules: information, motivation, and behavioral skills. Controls received no intervention. Preintervention data were subjected to statistical comparison using an independent sample *t* test, Mann-Whitney *U* test, and chi-square test. Primary and secondary indicators were measured using the Internet Addiction Test (IAT), Chinese Treatment Self-Regulation Questionnaire, and Internet Control Self-Efficacy Questionnaire. To evaluate the intervention’s effects and identify pre- to postintervention changes, we used generalized linear mixed models and generalized estimating equations for analysis with SPSS software (version 23.0), considering statistical significance at α=.05. Participants were further stratified into mild and moderate to severe IA subgroups based on their baseline scores, and subgroup analyses were conducted to examine both the intervention effects within each stratum and the between-group differences across different addiction severity levels.

**Results:**

The intervention group showed a significant reduction in IA (*χ*^2^_1_=14.154; *P*<.001). This paper confirmed a substantial reduction in addiction levels after the intervention compared to the control group (adjusted mean change –4.531, 95% CI –7.281 to –1.781; *P*=.001), with no significant differences in internet use duration. Consistent findings were obtained in the subgroup analyses. A time-group interaction was identified for sleep duration (*P*<.001), with a notable increase observed in the intervention group (odds ratio 2.186, 95% CI 1.142-4.183; *P*=.02). No significant differences were observed between the groups in sleep quality and somatic-psychological symptoms. Consistent findings were observed in the subgroup analyses of the above studies. The intervention group showed increased motivation (adjusted mean change 4.283, 95% CI 0.804-7.763; *P*=.02) and behavioral skills (adjusted mean change 3.407, 95% CI 1.771-5.043; *P*<.001) after the intervention. Only behavioral skills were improved in the mild IA subgroup, whereas only motivation showed improvement in the moderate to severe subgroup.

**Conclusions:**

The WeChat public-account intervention based on the IMB model significantly reduced the level of IA in college students and simultaneously had a positive effect on increasing sleep duration. The decline in IAT scores observed in the moderate to severe subgroup was twice as large as that in the mild subgroup.

**Trial Registration:**

ClinicalTrials.gov NCT06704984; https://clinicaltrials.gov/study/NCT06704984

## Introduction

The extensive and pervasive adoption of the internet across various societal sectors has resulted in a significant increase in the global number of internet users. According to the 53rd China Statistical Report on Internet Development released by China Internet Network Information Center [1], by December 2023, China had an internet user population of 1.092 billion, with a penetration rate of 77.5%. Remarkably, users spend an average of 26.1 hours online weekly, with 99.9% accessing the internet through mobile devices. This digital revolution has, however, introduced a significant societal challenge: internet addiction (IA), which is particularly concerning among adolescents and young adults [2]. IA, also termed internet addiction disorder, problematic internet use, compulsive internet use, and excessive internet use, refers to uncontrolled, impulsive internet use behavior without dependence on addictive substances. It is characterized by obvious impairments in academic performance, occupational development, and social functioning caused by excessive internet engagement [3]. A growing body of research demonstrates that IA inflicts multifaceted harm on the physical, psychological, and social functioning of young people. Physiologically, it is associated with fatigue, sleep disturbances, impaired concentration, eating disorders, and weight gain [4-7]; prolonged use of electronic devices can also lead to computer vision syndrome [8,9], tenosynovitis of the fingers and wrists [10], as well as musculoskeletal pain and decreased motor coordination [11]. Psychologically, individuals with IA often exhibit introversion, sensitivity, social phobia, high levels of loneliness, and self-isolation [12]; violent video game addiction can reduce empathy and increase aggressive behaviors [13], and in severe cases, it may be comorbid with mental disorders such as depression, anxiety disorders, and attention-deficit/hyperactivity disorder [14,15]. In terms of social functioning, IA can lead to academic procrastination, declining academic performance, and even school dropout among students [16]; it is also accompanied by communication difficulties, emotional dysregulation, and impulsive behaviors [17], which in turn cause problems such as poor interpersonal relationships, negative thinking, increased loneliness, and reduced self-esteem [18]. Adolescents are a high-risk group for IA. A Chinese meta-analysis shows that the pooled prevalence of IA among Chinese adolescents is 10.3% [19]; globally, the average prevalence among European adolescents is 4.4%, while that in Asian countries is significantly higher, ranging from 8.1% to 26.5% [20]. Among them, college students, as a core component of the young adult population, who rely heavily on the internet for education, entertainment, and communication, are especially susceptible to its allure. Their increased independence, coupled with often insufficient self-control and a quest for self-expression, makes them particularly vulnerable to the negative effects of digital media [4,5]. Consequently, many college students find themselves exhibiting symptoms of IA, which can inflict detrimental impacts on their physical, mental, and social well-being [6-8,17]. Studies worldwide confirm these concerns, with high IA prevalence rates among college students. For instance, Salarvand et al [21] found a 31.51% addiction rate among 16,585 Iranian college students. A meta-analysis involving 22 studies in 7 countries showed that the prevalence of IA among middle school students and college students in Africa was 32.2% and 42.0% [22]. Within China, Cai et al [23] conducted a study among 1070 medical students from 5 universities, discovering a prevalence range of 20.7% to 25.8% for IA, with senior students being more prone to this addiction than their junior counterparts. Given these alarming statistics and the profound implications of IA on college students’ lives, it is imperative that we take proactive measures to prevent and mitigate its onset.

Over the years, domestic and foreign scholars in different fields have carried out a large number of studies on the intervention methods of IA, mainly including group counseling [24], cognitive behavioral therapy [25], psychological health education [26,27], sports therapy [28], acupuncture [29], pharmacotherapy [30], and comprehensive intervention [31,32]. However, these interventions face significant challenges in scalability and accessibility due to constraints posed by time, space, participant numbers, cost, and privacy concerns. The emergence of online interventions for IA represents a groundbreaking approach, harmoniously blending psychological insights with internet-based information and communication technologies. This innovative model offers distinct advantages over conventional methods, including cost-effectiveness and broader accessibility, thus reaching a wider audience. It also overcomes barriers related to time, scheduling, location, and participant limits, promoting greater inclusivity [33]. In comparison to conventional face-to-face interventions, online interventions offer a unique advantage in mitigating the negative psychosocial impacts associated with stigma and social anxiety. They encourage individuals afflicted with IA to embrace help-seeking behavior, fueling social engagement and motivation [34,35]. Based on WeChat’s social influence and huge user scale, it is of great practical significance to carry out science and information communication through WeChat public accounts. Previous studies have shown that WeChat interventions have achieved certain effects in reducing tobacco [36] use and alcohol overuse [37], as well as in cardiovascular care, including blood pressure management [38], cardiac rehabilitation [39], and quality-of-life improvement in patients after atrial fibrillation catheter ablation [40]. Although research directly addressing IA through WeChat public account interventions is yet to be reported, mounting evidence from psychology and neurobiology underscores similarities between IA and other forms of substance and behavioral addictions [41]. This underlines the plausibility of online intervention as a viable and potentially impactful approach to ameliorate IA among college students.

The information, motivation, behavioral skills (IMB) model is mainly used to predict preventive health behaviors and implement health education. This model introduces the concepts of “motivation” and “self-efficacy” from rational behavior theory [42] and social cognition theory [43], respectively, and divides the factors affecting individual behavior transformation into 3 components: information, motivation, and behavioral skills. By using these 3 core components, the IMB model endeavors to elucidate the intricate process of behavior change, elucidating both the direct and indirect relationships among behavioral determinants, their strengths, and the directionality of these associations. Ultimately, this approach facilitates a deeper understanding and prediction of behaviors, guiding the exploration of optimal intervention strategies. Notably, information, motivation, behavior, and skills not only function as independent variables that directly shape individual behavior but also interactively influence behavior through their intricate interplay [44]. When individuals attain a threshold level of proficiency in these areas, they are poised to embark on behavioral shifts that ultimately contribute to health promotion goals. As a systematic and scientific theoretical model of behavioral transformation, the IMB model has been widely used in behavioral intervention studies of various diseases such as diabetes [45], AIDS [46], and cardiovascular disease [47]. Compared with other theories, the IMB model is easier to implement intervention practice from theoretical research, which can provide theoretical support for better prevention and control of IA.

Therefore, college students with IA were selected as the research participants in this study, and a 6-week randomized controlled trial was then conducted. This trial was designed to evaluate the effectiveness of a WeChat public account-based online intervention developed strictly according to the IMB model in reducing IA among college students. It also aimed to explore the potential mechanisms underlying addictive behavior change from the perspectives of information, motivation, and behavioral skills. The innovation of this study lies in integrating the IMB model with the WeChat public account platform to systematically construct an intervention program across the 3 dimensions of information, motivation, and behavioral skills. By taking advantage of the convenience and accessibility of online platforms, this study provides a more targeted and scalable practical approach for IA intervention among college students and offers a reference for developing a new model of online intervention for IA.

## Methods

### Overview

This study followed the reporting guidelines, which were established by CONSORT (Consolidated Standards of Reporting Trials; Multimedia Appendix 1) 2010 [48]. We are registered with ClinicalTrials.gov, registration number NCT06704984. The recruitment of participants for this study commenced on March 1, 2023, and concluded on April 1, 2023. Undergraduate students from a university in Anhui Province, China, were selected as the research participants in this study and were recruited through multiple on-campus channels. Recruitment notices were released online via campus web pages, official websites, and mainstream media platforms, while offline recruitment used posters, promotions in student activity areas, unified class notifications, and information dissemination among students. In the early phase, a survey and scale measurement were administered to all students at the university, and individual participation invitations were sent to those meeting the study’s inclusion and exclusion criteria. All participants signed informed consent prior to enrollment. Participants were randomized into intervention and control groups via gender-stratified randomization, with arbitrary cross-group transfer prohibited after enrollment.

### Ethics Approval

This study was approved by the Biomedical Ethics Committee of Anhui Medical University (approval number: 20190495). We are registered with ClinicalTrials.gov, registration number NCT06704984. All study procedures were conducted in strict accordance with the ethical principles of the Declaration of Helsinki. Written informed consent was obtained from all eligible participants prior to enrollment. All participants were fully informed of the study purpose, procedures, risks and benefits, and their right to withdraw from the study at any time without adverse consequences.

All study data were processed with full anonymization. No personally identifiable information (including full names, student ID numbers or contact details) was collected during the survey, and all participants were identified exclusively by unique numerical study IDs. Raw research data are stored in encrypted form on a dedicated secure server at the School of Public Health, Anhui Medical University, and are accessible only to authorized members of the research team. All study results are presented in aggregated statistical form, and no individual participant information will be disclosed in any publication.

All participants provided informed consent. Deidentified data were used for the whole analysis. Financial incentives (US $3.34 electronic cash) were distributed to those who completed the trial.

### Participant Eligibility Criteria

Textbox 1 presents the inclusion and exclusion criteria for this study.

**Eligibility criteria.**

**Inclusion criteria**

- College students diagnosed with internet addiction, as evidenced by an Internet Addiction Test scale score of 40 or above
- Age range between 18 and 24 years
- Use WeChat software
- Good physical health, capable of completing the whole process of the study
- Voluntary participation with a signed informed consent form

**Exclusion criteria**

- Presence of any mental illness or cognitive impairment
- Current or impending receipt of psychological or pharmacological treatment for a mental health condition
- Circumstances that would hinder participation, such as internships, suspensions, or other reasons for absence from school
- Refusal to participate in the study

### Randomization

Stratified randomization was adopted to generate the random allocation sequence for all participants. Participant recruitment was performed by dedicated research staff, and eligible participants were assigned to either the intervention or control group in strict accordance with the established randomized sequence. All enrolled participants were prohibited from switching groups throughout the study period to preserve the integrity of the study design. Given the inherent characteristics of the intervention program, participant blinding was not feasible in this study. To ensure methodological rigor and avoid potential bias, the roles of study participant recruitment, intervention implementation, and data collection were strictly separated. In addition, all statistical analyses were independently conducted by specialized statistical staff who were not involved in participant enrollment and intervention delivery.

### Sample Size Calculation

This study was designed as a randomized controlled trial, with sample size determination grounded in IA scores. Using a bilateral test with α=.05 and 1-β=.90, a 1:1 ratio was established between the intervention and control groups. Given the novelty of the WeChat public account intervention for IA, a preliminary trial involving 60 college students with the condition was conducted to inform sample size calculations. Participants were randomly assigned to either the intervention group (n=30) or the control group (n=30), yielding average Internet Addiction Test (IAT) scale scores of 50.27 (SD 12.28) in the intervention group and 56.43 (SD 12.64) in the control group. Based on these findings, a total sample size of 170 individuals was determined, with an allowance for 10% attrition due to ineligibility, noncompliance, or loss to follow-up, resulting in a requirement for 187 participants. Ultimately, 226 participants were recruited, with 113 assigned to each group.

### Interventions

The intervention group received a 6-week intervention leveraging an online WeChat public account that integrated the IMB model into its design. Throughout the intervention phase, participants received 2 posts every 2 days on the WeChat public account, with an additional post on weekends during the second to fifth weeks, totaling 46 interventions. These interventions were categorized into 11 focused on information, 19 on motivation, and 16 on behavioral skills. The intervention format included video, pictures, and text. Throughout the intervention period, engagement was monitored by tracking the readership of the sent content through the WeChat public account’s backend data. The intervention plan of the WeChat public account based on the IMB model is shown in Table S1 in Multimedia Appendix 2.

Participants in the control group received no intervention and were instructed to maintain a normal life and study state.

To mitigate potential contamination between the intervention and control groups, participants were instructed to refrain from sharing group-specific information with others during the intervention period. Furthermore, the WeChat public account was configured with search restrictions, ensuring that nonintervention participants could not access its content. Additionally, the researchers used the WeChat public account platform to monitor and confirm that only those in the intervention group were following the account, thereby facilitating the seamless execution of the intervention experiment.

### Data Collection and Outcomes

The researchers adopted a comprehensive approach to data collection, using paper questionnaires to gather both baseline demographic information and various indicators from participants before and 1 week after intervention.

#### Primary Indicators (IA)

The 20-item IAT scale prepared by Moon et al [49] was used to investigate the IA of the participants. Notably, the scale demonstrated robust reliability, with Cronbach α coefficients of 0.81 and 0.87 pre- and postintervention, respectively, and a retest reliability of 0.88.

#### Secondary Indicators

The participants’ internet time, sleep duration, sleep quality, and somatic-psychological symptoms in the past month were collected.

#### Other Indicators

##### Knowledge Related to IA (Information)

To assess participants’ knowledge regarding IA, a self-designed questionnaire was used, tailored from the comprehensive Knowledge and Attitude of Internet Addiction questionnaire by Huang [50]. This instrument comprises 10 judgment questions, ensuring a balanced assessment. The total score ranges from 0 to 10, reflecting a higher level of mastery over IA-related knowledge with an increased score. Notably, the questionnaire’s retest reliability of 0.84 underscores its high stability and reliability.

##### IA Improves Motivation

To gauge participants’ motivation to reduce their IA, the Treatment Self-Regulation Questionnaire was used, drawing upon the Chinese version adapted by Zhou and Li [51] in collaboration with foreign scholars. Higher scores indicative of a stronger willingness to engage in treatment for IA demonstrate the instrument’s sensitivity to individual motivation levels. The questionnaire’s Cronbach α coefficient, both pre- and postintervention, stood at 0.81, while its retest reliability reached 0.82, reinforcing its internal consistency and reliability.

##### Internet Control Self-Efficacy (Behavioral Skills)

To evaluate participants’ behavioral skills in managing their IA, the Internet Control Self-Efficacy Questionnaire compiled by Luo et al [52] was administered. The higher the score, the higher the network control self-efficacy. In this study, the Cronbach α coefficients of the scale before and after intervention were 0.90 and 0.93, respectively, and the retest reliability was 0.80.

### Statistical Analysis

For the continuous variables, a comprehensive assessment of the changes in IAT scale scores, time spent online, and IMB model scores between the intervention and control groups, both pre- and postintervention, was conducted using generalized linear mixed models. This approach allowed us to account for potential variability within and between participants, enhancing the robustness of our findings. Specifically, the model was constructed with fixed effects comprising group factors, time factors, and their interaction (group × time), providing insights into how the intervention impacted these outcomes over time. Holm-Bonferroni correction was used to compare the differences between groups at different time points on the basis of corrected baseline data. For categorical variables, the differences in sleep duration, sleep quality, and somatic-psychological symptoms between the 2 groups before and after intervention were analyzed by generalized estimating equation. Group factors, time factors, and group × time interaction terms were incorporated into the model to compare the differences between groups. When the outcome variables were ordered multiple categorical variables (sleep duration and sleep quality), the difference between the groups at different time points was analyzed by ordered logistic regression. Meanwhile, all participants were divided into mild and moderate-severe IA subgroups based on baseline IAT scores to explore differences in intervention effects across subgroups (bilateral test, α=.05).

### Quality Control

To guarantee research quality and minimize bias, the trial followed CONSORT 2010 with full-process quality control spanning design, implementation, and data management. Predefined eligibility criteria and unified IAT screening restricted enrollment to qualified participants to lower selection bias.

All researchers completed standardized prestudy training covering trial aims, interventions, and survey protocols. Standardized guidance, on-site monitoring, and spot checks unified questionnaire administration to mitigate operational bias.

Participants were allocated via stratified randomization for balanced grouping and reduced allocation bias. Raw questionnaires were inspected for integrity, alongside double data entry and cross-checking to secure data quality.

## Results

### Baseline Characteristics of the Participants

**Figure 1:**
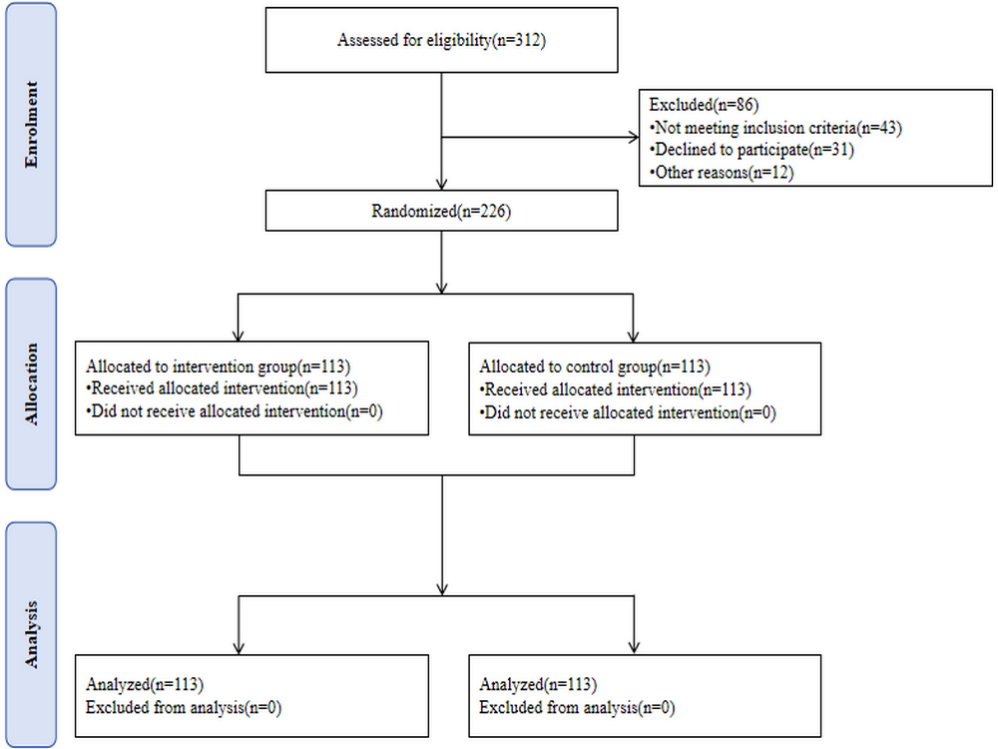
CONSORT (Consolidated Standards of Reporting Trials) flow diagram.

A total of 226 participants, who fulfilled the inclusion criteria, were chosen from a pool of 312 college students. Stratified by gender, they were randomly assigned to either the intervention group (n=113) or the control group (n=113), as depicted in the CONSORT flowchart in Figure 1. No statistically significant differences were observed in terms of age, gender, family residence, or the status of being an only child (*P*>.05). At the outset of the study, participants exhibited an average IAT score of 53.43 (SD 9.99), and the mean daily internet use over the past month was 7.76 (SD 2.54) hours. A thorough statistical comparison of key outcome indicators—including IAT scores, internet use duration, sleep duration, sleep quality, and the presence of somatic and psychological symptoms, as well as IMB model scores—between the intervention and control groups showed no significant discrepancies (*P*>.05). Collectively, participants in the 2 groups presented balanced demographic profiles and consistent baseline outcome levels, establishing a reliable premise for subsequent intervention effect analysis. Detailed comparisons of demographic characteristics and subgroup baseline indicators between the 2 groups are presented in Table 1.

**Table 1:** Comparison of baseline characteristics between the 2 groups.

Variables											Total sample size (n=226)		Intervention group (n=113)		Control group (n=113)		Statistical value			*P* value	
Age (years), mean (SD)											20.75 (0.93)		20.79 (1.02)		20.71 (0.82)		–0.646 (224)^a^			.52	
**Gender, n (%)**																		0.001 (1)^b^			>.99
							Male				110 (48.7)		55 (48.7)		55 (48.7)						
							Female				116 (51.3)		58 (51.3)		58 (51.3)						
**Family place of residence, n (%)**																		1.220 (3)^b^			.75
									Village	79 (35)		38 (33.6)		41 (36.3)							
									Township	38 (16.8)		17 (15)		21 (18.6)							
									County	54 (23.9)		30 (26.5)		24 (21.2)							
									City	55 (24.3)		28 (24.8)		27 (23.9)							
**Only** **child, n (%)**																		2.304 (1)^b^			.13
				Yes						83 (36.7)		47 (41.6)		36 (31.9)							
				No						143 (63.3)		66 (58.4)		77 (68.1)							
**Education level of father, n (%)**																		1.441 (4)^b^			.84
						Primary or below				39 (17.3)		22 (19.5)		17 (15)							
						Junior high school				87 (38.5)		40 (35.4)		47 (41.6)							
						Senior high school				24 (10.6)		13 (11.5)		11 (9.7)							
						Technical/technical school				19 (8.4)		9 (8)		10 (8.8)							
						College or above				57 (25.2)		29 (25.7)		28 (24.8)							
**Education level of mother, n (%)**																		3.930 (4)^b^			.42
			Primary or below							81 (35.8)		37 (32.7)		44 (38.9)							
			Junior high school							62 (27.4)		33 (29.2)		29 (25.7)							
			Senior high school							24 (10.6)		9 (8)		15 (13.3)							
			Technical/technical school							24 (10.6)		13 (11.5)		11 (9.7)							
			College or above							35 (15.5)		21 (18.6)		14 (12.4)							
**Annual household income per capita (1 yuan=US** **$0.15),** **n (%)**																		6.891 (4)^b^			.14
		<10,000 yuan								38 (16.8)		13 (11.5)		25 (22.1)							
		10,000 to 19,900 yuan								49 (21.7)		27 (23.9)		22 (19.5)							
		20,000 to 29,900 yuan								40 (17.7)		25 (22.1)		15 (13.3)							
		30,000 to 39,900 yuan								31 (13.7)		15 (13.3)		16 (14.2)							
		≥40,000 yuan								68 (30.1)		33 (29.2)		35 (31)							
**Academic performance, n (%)**																		0.852 (2)^b^			.65
	Lower middle class/lower class									53 (23.5)		29 (25.7)		24 (21.2)							
	Intermediate									75 (33.2)		38 (33.6)		37 (32.7)							
	Medium/good									98 (43.4)		46 (40.7)		52 (46)							
IAT^d^ scale, mean (SD)											53.43 (9.99)		53.66 (11.03)		53.20 (8.88)		–0.346 (224)^a^			.73	
Internet use (hours), mean (SD)											7.76 (2.54)		8.01 (2.50)		7.52 (2.57)		–1.461 (224)^a^			.15	
**Sleep time, n (%)**																		5.34 (2)^b^			.07
								≤6 hours		25 (11.1)		13 (11.5)		12 (10.6)							
								6.01-8.00 hours		129 (57.1)		72 (63.7)		57 (50.4)							
								>8 hours		72 (31.9)		28 (24.8)		44 (38.9)							
**Sleep quality, n (%)**																		1.309 (2)^b^			.52
						Good				65 (28.8)		30 (26.5)		35 (31)							
						Normal				142 (62.8)		75 (66.4)		67 (59.3)							
						Poor				19 (8.4)		8 (7.1)		11 (9.7)							
**Somatopsychological symptoms, n (%)**																					
					Dizziness (yes)					43 (19)		23 (20.4)		20 (17.7)		0.258 (1)^b^			.61		
					Eye discomfort (yes)					95 (42)		45 (39.8)		50 (44.2)		0.454 (1)^b^			.50		
					Gastrointestinal problems (yes)					57 (25.2)		32 (28.3)		25 (22.1)		1.150 (1)^b^			.28		
					Depressed mood (yes)					49 (21.7)		28 (24.8)		21 (18.6)		1.277 (1)^b^			.26		
					Anxiety (yes)					67 (29.6)		38 (33.6)		29 (25.7)		1.718 (1)^b^			.19		
					Difficulty concentrating (yes)					61 (27)		37 (32.7)		24 (21.2)		3.795 (1)^b^			.05		
**IMB** ^e^ **model score**																					
				Information, median (IQR)						10.00 (9.00-10.00)		10.00 (9.00-10.00)		10.00 (9.00-10.00)		–0.091^c^			.93		
				Motivation, mean (SD)						69.27 (14.11)		68.67 (13.65)		69.88 (14.59)		–0.640 (224)^a^			.52		
				Behavior skills, mean (SD)						29.40 (5.94)		29.40 (5.80)		29.41 (6.10)		0.011 (224)^a^			.99		

^a^*t* test (*df*).

^b^Chi-square (*df*).

^c^Mann-Whitney *U* test.

^d^IAT: Internet Addiction Test.

^e^IMB: information, motivation, behavioral skills.

### Intervention Effect of WeChat Public Account Intervention on IA of College Students

#### Primary Indicators

We categorized the variations in IAT scores before and after the intervention into 2 distinct groups: a reduction of ≥12 points in the IAT score was deemed indicative of a significant improvement in IA symptoms, whereas any lesser decrease was considered as no substantial improvement. Notably, the chi-square test yielded compelling results, revealing a statistically significant divergence in the intervention effects between the 2 groups (*χ*^2^_1_=14.154; *P*<.001), as tabulated in Table 2.

**Table 2:** Comparison of internet addiction intervention effectiveness between groups.

Subgroup and groups			Effective, n (%)		Not effective, n (%)		Chi-square (*df*)			*P* value	
**All sample**								14.154 (1)			<.001
	Intervention group	32 (28.3)		81 (71.7)							
	Control group	10 (8.8)		103 (91.2)							
**Mild internet addiction**								8.831 (1)			.003
	Intervention group	24 (23.8)		77 (76.2)							
	Control group	9 (8.6)		96 (91.4)							
**Moderate to severe internet** **addiction** ^a^								—^b^			.03
	Intervention group	8 (66.7)		4 (33.3)							
	Control group	1 (12.5)		7 (87.5)							

^a^In the moderate to severe internet addiction subgroup, the expected cell counts were less than 5 in 50% of cells, failing to meet the chi-square test criteria. The Fisher exact test was adopted instead.

^b^Not available.

This analysis unveiled a marked intergroup interaction effect on the IAT scores, achieving statistical significance (*P*<.001). Importantly, both groups experienced a decline in their IAT scores after the intervention, with the intervention group exhibiting a notably more pronounced reduction compared to the control group. This consistent trend was also observed in subgroup analyses. (Table 3 and Table S2 in Multimedia Appendix 2) IAT scores at different time points between groups were further analyzed, and the results are shown in Table 4. Intergroup comparisons after adjusting for baseline showed a statistically significant decrease in IAT scores in the intervention group compared with the control group (adjusted mean change –4.531, 95% CI –7.281 to –1.781; *P*=.001)

**Table 3:** Results of the generalized linear mixed effects model analysis (control group: n=113; intervention group: n=113).

Group			Baseline	Postintervention	Interaction term, *P* value	Group effect, *P* value		Time effect, *P* value	
**IAT** ^a^ **scores, mean (SD)**					<.001		.10		<.001
	Intervention group		53.66 (11.03)	47.88 (11.16)					
	Control group		53.20 (8.88)	52.41 (10.85)					
**Internet** **use (hours)** **, mean (SD)**					.008		.90		<.001
	Intervention group		8.01 (2.50)	6.36 (2.60)					
	Control group		7.52 (2.57)	6.93 (2.76)					
**IMB** ^b^ **model scores**									
	**Information, median (IQR)**				.28		.76		.02
		Intervention group	10.00 (9.00-10.00)	10.00 (9.00-10.00)					
		Control group	10.00 (9.00-10.00)	10.00 (9.00-10.00)					
	**Motivation, mean (SD)**				.001		.33		<.001
		Intervention group	68.67 (13.65)	74.65 (12.22)					
		Control group	69.88 (14.59)	70.36 (12.65)					
	**Behavioral skills, mean (SD)**				<.001		.02		<.001
		Intervention group	29.40 (5.80)	32.82 (6.90)					
		Control group	29.41 (6.10)	29.42 (6.17)					

^a^In the moderate to severe internet addiction subgroup, the expected cell counts were less than 5 in 50% of cells, failing to meet the chi-square test criteria. The Fisher exact test was adopted instead.

^b^Not available.

**Table 4:** Mean change from baseline in Internet Addiction Test (IAT) scores, internet use time, and information, motivation, behavioral skills (IMB) model scores; reported values are posttest mean changes comparing the intervention group with the control group (reference).

Subgroup and groups				Mean change (95% CI)		*P* value^a^
**Overall** **sample**						
	IAT score		–4.531 (–7.281 to –1.781)		.001	
	Internet use (hours)		–0.565 (–1.247 to 0.117)		.10	
	**IMB model scores**					
		Motivation	4.283 (0.804 to 7.763)		.02	
		Behavioral skills	3.407 (1.771 to 5.043)		<.001	
**Mild internet addiction**						
	IAT score		–4.657 (–7.121 to –2.193)		<.001	
	Internet use (hours)		–0.677 (–1.365 to 0.011)		.05	
	**IMB model scores**					
		Motivation	1.707 (–1.354 to 4.769)		.27	
		Behavioral skills	3.163 (1.468 to 4.857)		<.001	
**Moderate to severe internet addiction**						
	IAT score		–9.292 (–17.724 to –0.859)		.03	
	Internet use (hours)		–0.979 (–3.681 to 1.724)		.47	
	**IMB model scores**					
		Motivation	15.635 (4.829 to 26.412)		.006	
		Behavioral skills	5.308 (–1.161 to 11.578)		.11	

^a^IAT: Internet Addiction Test.

^b^IMB: information, motivation, behavioral skills.

#### Secondary Indicators

##### Changes in Internet Use Time

The results showed that there was a significant interaction effect between groups (*P*=.008). Notably, after the intervention, both the intervention and control groups exhibited a reduction in their internet use time. However, the decrease observed within the intervention group was notably more pronounced compared to the control group. This consistent trend was also observed in subgroup analyses (Table 3 and Table S2 in Multimedia Appendix 2). When comparing groups after adjusting the baseline, no significant difference between groups was observed (*P*=.10), as shown in Table 4.

##### Changes in Sleep Duration

Sleep duration as a categorical variable was taken as the dependent variable (1=≤6 hours; 2=6~8 hours; 3=>8 hours). The paper revealed a statistically significant interaction effect between the groups (*P*<.001). This consistent trend was also observed in subgroup analyses. (Tables S3 and S4 in Multimedia Appendix 2)

Prior to the intervention, the comparison between groups failed to yield a statistically significant difference in sleep duration (*P*=.05). However, after the intervention, a marked statistical difference emerged (*P*=.02), with an odds ratio of 2.186 (95% CI 1.142-4.183). This suggests that, compared to the control group, the intervention group experienced a notably longer sleep duration after the intervention. In other words, the intervention group had a 2.186 times higher likelihood of sleeping longer than the control group, as detailed in Table 5. In the mild IA subgroup, participants in the intervention group were 2.050 times more likely to achieve longer sleep duration after treatment. By contrast, no significant intergroup discrepancy was observed in the moderate to severe IA subgroup, with no remarkable elevation in the probability of prolonged sleep duration.

**Table 5:** Results of ordered logistic regression analyses of sleep duration; reported β, odds ratio (OR), and *P* value refer to the intervention group compared with the control group (reference: OR 1.000).

Subgroup, time, and groups			β		OR (95% CI)		*P* value
**Overall sample**							
	Baseline	–0.512		0.599 (0.357-1.005)		.05	
	Postintervention	0.782		2.186 (1.142-4.183)		.02	
**Mild internet addiction**							
	Baseline	–0.661		0.516 (0.299-0.891)		.02	
	Postintervention	0.718		2.050 (1.042-4.035)		.04	
**Moderate to severe internet addiction**							
	Baseline	1.274		3.575 (0.515-24.854)		.20	
	Postintervention	1.529		4.614 (0.407-52.353)		.22	

^a^*P* value after Holm-Bonferroni correction.

##### Sleep Quality

The generalized estimating equation model analysis showed that there was no significant interaction effect between groups in the difference in sleep quality. (Tables S3 and S4 in Multimedia Appendix 2)

##### Somatic-Psychological Symptoms

**Figure 2:**
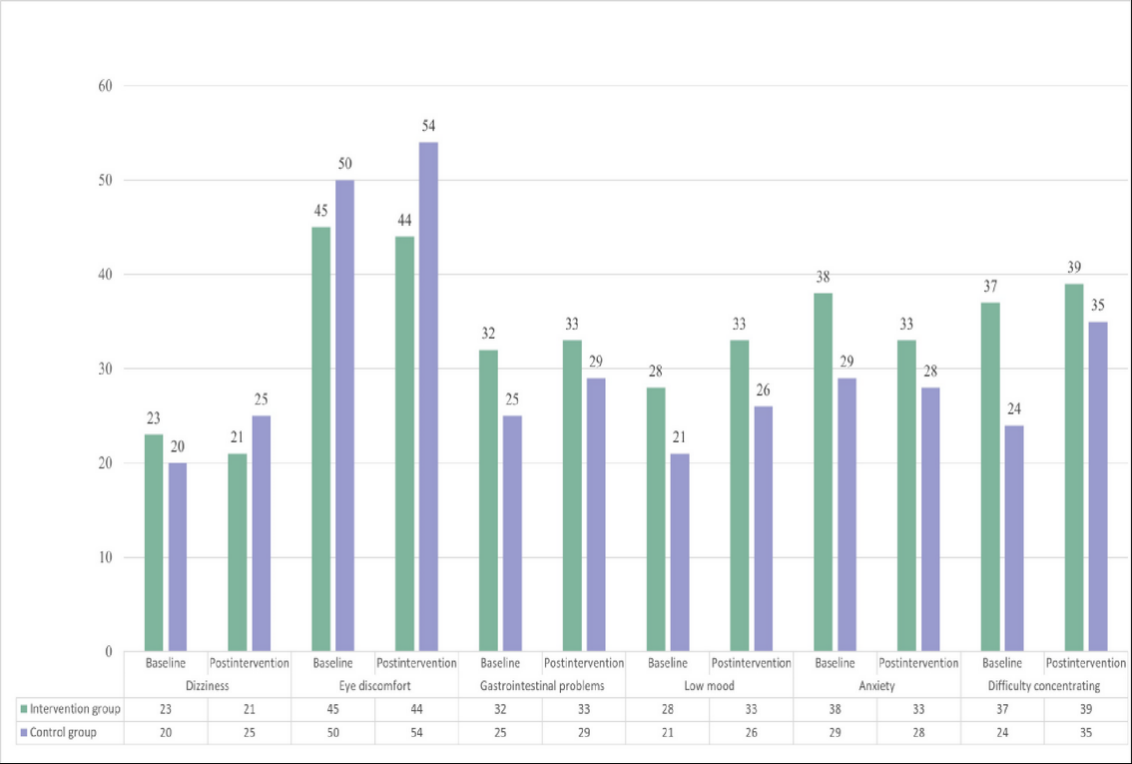
Somatic-psychological symptoms pre- and postintervention.

Figure 2 illustrates that the number of individuals reporting dizziness, eye discomfort, and anxiety symptoms decreased following the intervention. Conversely, an increase in the number of participants reporting other symptoms was observed. Generalized estimating equation analysis indicated that binary classification of somatic-psychological symptoms was set as the dependent variable, with group, time, and group × time interaction as predictors for intervention effect analysis. No significant group-by-time interaction or intergroup differences were found for all somatic-psychological symptoms (Table S3 in Multimedia Appendix 2).

#### Other Indicators

During the intervention, the average reading counts of WeChat-delivered intervention content across 3 categories were tracked via the WeChat official account backend. The average reading count was highest for motivation content (n=89), followed by behavior skills content (n=81), and information content (n=73). Generalized linear mixed models were used to meticulously examine the influence of group and time factors on IMB model scores. The results revealed significant intergroup interaction effects for both motivation score (*P*=.001) and behavioral skill score (*P*<.001). Specifically, after the intervention, the intervention group exhibited notable enhancements in both motivation and behavioral skills compared to their preintervention levels, as indicated in Table 3.

Upon further intergroup comparison, adjustments were made to account for baseline variations. The adjusted findings highlighted a statistically significant increase in the motivation score of the intervention group after the intervention, with a marked difference from the control group (adjusted mean change 4.283, 95% CI 0.804-7.763; *P*=.02). Similarly, the behavioral skill scores of the intervention group surpassed those of the control group after the intervention, with the disparity reaching statistical significance (adjusted mean change 3.407, 95% CI 1.771-5.043; *P*<.001; Table 4).

## Discussion

### Principal Findings

This randomized controlled trial investigates the efficacy of an IMB model–based WeChat public-account intervention in reducing IA among college students and its impact on sleep duration. The study indicated that the intervention significantly decreased IA levels and positively affected sleep duration. Moreover, a significant improvement in sleep duration was observed in the mild IA subgroup after intervention. It did not reduce internet use time, improve sleep quality, or alleviate somatic-psychological symptoms of internet-addicted college students.

This study demonstrated that following the intervention, no significant differences were observed in information scores between the intervention group and the control group. Participants in both groups exhibited equivalent levels of knowledge regarding IA. This suggests that merely acquiring information about withdrawal from IA is not sufficient to directly reduce the severity of IA. Individual behavior change is a multifaceted process influenced by a variety of factors. According to the IMB model, individuals are more likely to achieve behavior change and meet their desired goals by creating an environment that fosters the transformation of personal attitudes, enhancing motivation for behavior change, and providing specific behavioral skills, particularly by improving self-efficacy [53]. Intervention research based on this model has addressed the traditional imbalance of “the knowledge-behavior gap,” placing greater emphasis on enhancing motivation and improving behavioral skills, which ultimately enhances the effectiveness of interventions [54]. Specifically, the marked increase in motivation scores observed within the intervention group, both intragroup and when compared to the control group, indicates that the intervention of WeChat public account enhanced the motivation of the study participants to reduce IA. Only motivation showed improvement in the moderate to severe IA subgroup. This is also supported by the findings of Bottel et al [55] that online intervention can significantly reduce the severity of IA and duration of internet use by increasing the motivation to abstain from internet use behavior. Similarly, Niemann et al [56] conducted an intervention study involving 169 participants, showing that an online-based incentive intervention program can effectively promote motivation to change behavior, thereby reducing problematic internet use and preventing its progression to IA. These results align with our study, highlighting the critical role of motivation in reducing IA.

The IMB model categorizes improvement motivation into 2 distinct aspects: individual motivation and social motivation. In terms of individual motivation, enhancing individuals’ attitudes, beliefs, and perceived benefits associated with quitting IA can effectively stimulate their drive to actively pursue change, thereby facilitating the goal of reducing IA. The efficacy of motivational enhancement therapy, which revolves around eliciting ambivalence toward cessation (withdrawal motivation) and impeding maintenance tendencies (approach motivation), has garnered significant attention within the research community. Its application across diverse addiction disorders, including IA, gambling, alcoholism, and smoking, has demonstrated remarkable interventional outcomes [57,58]. In terms of social motivation, the social support obtained by encouraging individuals to quit IA may help reduce the level of IA [59]. According to the cognitive behavioral model proposed by Davis [60], when there is a lack of adequate social support in real life, individuals may turn to the internet to find dynamic social support to meet their psychological needs. Enter the WeChat public account intervention, an innovative, user-centered approach rooted in the IMB model and tailored specifically for individuals struggling with IA. This strategy harmoniously integrates the dual objectives of enhancing individual motivation for change and fostering social support, aligning with the principles of motivational interviewing as conceptualized by Miller and Rollnick [61]. Motivational interviewing is a collaborative conversational technique designed to amplify an individual’s intrinsic motivation and commitment toward positive change [62]. Building on this foundation, this study leverages the WeChat platform to integrate the motivation to reduce IA into the intervention plan. The entire intervention process is noncoercive and voluntary, making it particularly suitable for those who are reluctant or ambivalent about actively quitting IA due to feelings of shame.

In terms of behavioral skills, the results of this study reveal that following the intervention, the behavioral skills scores of participants in the intervention group were significantly higher than that of the control group. Only behavioral skills were improved in the mild IA subgroup. This indicates that the intervention delivered via the WeChat public account effectively enhanced the self-efficacy of individuals with IA. A randomized controlled trial conducted by Dieris-Hirche et al [63] showed that online intervention can reduce symptoms of IA while increasing participants’ motivation to change and self-efficacy, which is consistent with the findings of this study. Relevant studies have pointed out that conventional health education still relies on passive supervision and spoon-feeding. In this mode, patients have a low level of self-efficacy, lack the autonomy to understand knowledge, and are prone to negative emotions, which makes the intervention effect unsatisfactory [64]. The health education intervention mode of a WeChat public account in this study can provided continuous health information and promoted self-efficacy among individuals with IA regarding personal motivation. Studies have found that the application of reading therapy can effectively reduce a low degree of IA among college students [65]. Therefore, we also used videos, text, pictures, and other forms to motivate the study participants to reduce their IA behavior by increasing outdoor activities, physical exercise, finding alternative hobbies, downloading mobile phone management software. Second, motivational intervention based on the IMB model makes individuals with IA realize that unhealthy behaviors or noncompliance with the intervention may bring serious consequences, which can promote the change of treatment attitude of the study participants, thus strengthening self-efficacy. Through the typical case sharing function of the platform, the persuasion of health education information can be enhanced in the form of presentation, and peer effects can be generated to further improve the intervention effect.

Despite the lack of significant differences in internet use time between the groups, this finding may be explained by the unique nature of the intervention delivered through WeChat public accounts. Participants were required to access the internet to obtain intervention tools, receive intervention content, and engage in online communications with researchers. This necessity likely sustained their overall online presence. Consequently, although the intervention may have enhanced participants’ attitudes toward reducing IA, the immediate reduction in online time may not be readily apparent. This aligns with previous findings in online intervention studies [66]. Academic pressure may be a contributing factor to the lack of significant change in internet use time. Research participants may need to use the internet to consult materials, prepare class notes, and participate in online learning. Although they continue to use electronic devices, the purpose and nature of their internet use have shifted. The internet is now primarily used for self-improvement and to meet academic needs, thereby reducing academic pressure.

Excessive internet use poses a multifaceted threat to both physical and mental well-being, encompassing issues such as compromised sleep quality, dizziness, ocular strain, unhealthy dietary habits, as well as symptoms of depression and anxiety [67,68]. Prolonged sleep deprivation among adolescents significantly impairs their emotional regulation capabilities, manifesting in heightened depression, apathy, anxiety, and fatigue [69]. Furthermore, the blue light emitted by electronic screens is believed to disrupt sleep patterns, with cumulative effects that can hinder the normal production of melatonin, delaying sleep onset and subsequently deteriorating sleep quality among individuals with IA [70]. These findings suggest the potential benefits of preventing and controlling IA for the healthy development of college students. Regarding sleep conditions, the intervention outcomes of this study reveal a noteworthy improvement. Specifically, participants in the intervention group, who received the WeChat public account–based IMB model intervention, had a significantly longer sleep duration compared to the control group, indicating a positive influence on enhancing sleep duration. However, no discernible differences were observed in sleep quality between the 2 groups. This lack of significant change in sleep quality could stem from several factors. First, individual sleep issues often stem from long-standing lifestyle habits, and the relatively brief 6-week intervention period in this study might not be sufficient to effect substantial improvements in sleep quality, suggesting that a longer-term intervention may be necessary to achieve optimal results. Second, the duration of sleep alone does not necessarily equate to its quality, as factors such as personal routines, sleep environments, sleep latency, and mental health status can significantly impact sleep quality [71,72]. In delving into the somatic-psychological symptomatology, this study failed to detect significant differences between the groups, which may be attributed to the intervention’s temporal scope. Given that somatic-psychological symptoms often emerge as a consequence of prolonged processes, their resolution necessitates a comprehensive examination of various factors, including individual traits, the depth of addiction, and the severity of symptoms. Consequently, a short-term intervention might not suffice to significantly mitigate these symptoms. In the future, it is necessary to further explore the long-term effect of WeChat public account intervention in improving the somatic-psychological symptoms of individuals with IA.

### Limitation

This study is undeniably constrained by several limitations that merit attention. First, the sampling framework is inherently limited, as participants were exclusively drawn from a single school, resulting in a potentially underrepresented sample. Future research should aim to broaden the participant pool to include a more diverse range of individuals, thereby enhancing the robustness and applicability of the results. Second, the intervention duration in this study was restricted to a mere 6 weeks. Such a short time frame may not be sufficient to fully capture the nuanced effects of the WeChat public account intervention. In addition, intervention adherence was assessed mainly using WeChat official account reading data, which is a relatively limited measure. Although it can indicate whether participants were exposed to the intervention materials, it cannot determine whether they read the content thoroughly and may therefore overestimate actual adherence. Future studies should use multiple indicators, such as reading duration, postreading quizzes, check-in records, and follow-up interviews, to provide a more comprehensive assessment of adherence. To address these limitations, future studies should conduct longer-term intervention trials with rigorous and systematic follow-up assessments to verify the effectiveness of the intervention and examine the stability and sustainability of its effects over time. In addition, a multidimensional adherence assessment system should be established by incorporating multiple indicators, such as reading duration, postreading quizzes, check-in records, and follow-up interviews, to provide a more comprehensive and accurate evaluation of intervention adherence.

### Future Prospects

While human-led IA intervention models can achieve personalized communication and content customization, they have inherent limitations such as high labor costs, limited service coverage, and difficulty in large-scale promotion. In recent years, groundbreaking advances in artificial intelligence (AI) technology in the field of chronic disease management have provided new insights to address this challenge. The Hyper-DREAM multimodal management platform and Cascade large language model agent developed by Wang et al [73,74] have confirmed that AI-powered digital phenotyping analysis, personalized recommendation systems, and intelligent consultation tools can significantly improve intervention efficiency, reduce service costs, and deliver standardized health management.

In the future, deep integration of AI technology with the IMB model should be prioritized. We can construct individual IA digital phenotypes via multimodal data collection to enable precise intervention matching, develop large language model–based intelligent consultation systems grounded in evidence-based psychological techniques to alleviate the shortage of professional personnel, and establish automated intervention effect evaluation and dynamic adjustment mechanisms. Ultimately, large-scale intervention platforms covering the entire care process can be built leveraging national-level applications such as WeChat, while rigorous attention should be paid to data privacy protection and algorithmic ethics, to provide practical digital solutions for IA prevention and control.

### Conclusion

A WeChat public-account intervention based on the IMB model can significantly reduce the level of IA among college students and has a certain positive effect on improving sleep duration. The effectiveness of the WeChat public-account intervention in reducing the amount of time spent online and improving sleep quality and somatic-psychological symptoms in college students with IA has not been found. In summary, the findings of this study can be used as a supplement to the existing research evidence of online intervention for IA, and they provide a reference for the intervention research of new IA.

## Data Availability

The datasets generated and analyzed during this study are available in the supplementary files submitted with this manuscript. All relevant data supporting the findings of this study are included within the paper and its supplementary materials. Additional information can be obtained from the corresponding author upon reasonable request.

## Appendix

Multimedia Appendix 1 CONSORT 2010 checklist.

Multimedia Appendix 2 WeChat public account intervention scheme based on the information, motivation, behavioral skills model and statistical analysis of its effects on internet addiction, sleep, and somatic-psychological symptoms.

## Abbreviations

AI: artificial intelligence
CONSORT: Consolidated Standards of Reporting Trials
IA: internet addiction
IAT: Internet Addiction Test
IMB: information, motivation, behavioral skills
