# Supplementary material for: Effectiveness of WeChat Public Account Intervention Based on the Information-Motivation-Behavioral Skills Model Among College Students With Internet Addiction: Randomized Controlled Trial
Source: J Med Internet Res. 2026 Jul 3;28:e84664. doi: 10.2196/84664 (PMC13379699; doi:10.2196/84664)
Supplement: Multimedia Appendix 2 [file jmir_v28i1e84664_app2.docx]

**Multimedia Appendix 2**

**Table S1** The intervention scheme of WeChat public accounts based on the IMB model

| Intervention topic | Specific content |
| --- | --- |
| Information | Disease knowledge introduction: including the prevalence of Internet addiction, common types, symptoms and risk factors that may aggravate addiction symptoms. |
|  | News dynamic: Update the latest news about Internet addiction in real time. |
|  | Real stories and experience sharing: Real cases of successful withdrawal from Internet addiction. |
|  | Treatment methods: Common treatment methods for Internet addiction are introduced, including psychological therapy, drug therapy, exercise intervention, music therapy, reading therapy and other intervention methods as well as treatment precautions, and the importance of compliance is emphasized. |
| Motivation | Self-monitoring: Dynamic assessment and recording of IAT scale scores to assess addiction levels in a timely manner. |
|  | Emotional management: Give psychological support to the research object, listen patiently, encourage the research object to express their ideas, communicate with them more, and objectively analyze the advantages and problems existing in the research object. Encourage research subjects and share success stories to build their confidence. |
|  | Information management: Provide guidance to the research subjects on corresponding addiction information, answer their doubts about Internet addiction information, inform the research subjects to pay more attention to the official information, and improve their ability to identify information. |
|  | Social support: The communication between subjects and staff and between subjects can resonate and increase their motivation to improve Internet addiction, thereby changing addictive behaviors. The staff provides individualized advice based on the main problems of the research subjects, discusses strategies to overcome the problems, and gives appropriate suggestions and feedback. By giving positive reinforcement to the subjects, the confidence of the subjects was effectively improved. |
| Behavior skills | Internet addiction prevention methods and healthy lifestyle: set goals, reasonable time management, find alternative hobbies, actively participate in outdoor activities, regular physical exercise, etc. |
|  | Targeted health education: The study subjects are guided according to the initial assessment to help the study subjects get rid of the addiction state. |
|  | Self-efficacy: According to the scale of self-assessment, actively participate in background messages, enhance self-efficacy. |
|  | Enhanced behavior: Feedback on the effects of the intervention, exchange of views with staff or study subjects. |
| Information-motivation-behavioral skills | Problem solving: re-evaluate the information, motivation and behavioral skills of the research subjects through exchange feedback, and push relevant content according to the existing problems of the research subjects. |

**Table S2** Results of subgroup analysis based on generalized linear mixed effects model

|  | Group | N | *M*（*P*_25_, *P*_75_)/$\bar{x}$±*s* | | *P* | | |
| --- | --- | --- | --- | --- | --- | --- | --- |
| Subgroup |  |  | Baseline | Postintervention | Interaction term | Group effect | Time effect |
| mild Internet addiction | IAT scores |  | | | | | |
|  | Intervention group | 101 | 51.05±8.08 | 46.51±10.04 | .12 | .09 | .26 |
|  | Control group | 105 | 51.77±7.44 | 51.17±10.12 |  |  |  |
|  | Internet usage time |  | | | | | |
|  | Intervention group | 101 | 7.87±2.52 | 6.38±2.49 | .18 | .51 | <.001 |
|  | Control group | 105 | 7.36±2.57 | 6.73±2.68 |  |  |  |
|  | IMB scores |  |  |  |  |  |  |
|  | Information |  |  |  | .33 | .57 | .02 |
|  | Intervention group | 101 | 9.06±1.38 | 9.34±1.20 |  |  |  |
|  | Control group | 105 | 9.24±1.07 | 9.32±0.88 |  |  |  |
|  | Motivation |  |  |  | <.001 | .90 | .003 |
|  | Intervention group | 101 | 63.14±11.08 | 67.82±10.38 |  |  |  |
|  | Control group | 105 | 66.76±11.71 | 66.11±11.45 |  |  |  |
|  | Behavioral skills |  |  |  | .001 | .02 | .01 |
|  | Intervention group | 101 | 29.73±5.67 | 32.52±6.83 |  |  |  |
|  | Control group | 105 | 29.54±6.05 | 29.36±6.14 |  |  |  |
| moderate to severe Internet addiction | IAT scores |  |  |  |  |  |  |
|  | Intervention group | 12 | 75.67±7.35 | 59.33±13.83 | .29 | .24 | .88 |
|  | Control group | 8 | 72.00±1.78 | 68.63±6.19 |  |  |  |
|  | Internet usage time |  |  |  |  |  |  |
|  | Intervention group | 12 | 9.22±2.11 | 6.18±3.50 | .30 | .53 | .34 |
|  | Control group | 8 | 9.53±1.40 | 9.52±2.56 |  |  |  |
|  | IMB scores |  |  |  |  |  |  |
|  | Information |  |  |  | .25 | .04 | .43 |
|  | Intervention group | 12 | 9.67±0.49 | 9.50±0.67 |  |  |  |
|  | Control group | 8 | 8.25±1.83 | 8.88±0.99 |  |  |  |
|  | Motivation |  |  | 63.38±15.85 | .024 | .08 | .49 |
|  | Intervention group | 12 | 76.42±7.38 | 79.00±9.15 |  |  |  |
|  | Control group | 8 | 66.50±15.21 | 8.88±0.99 |  |  |  |
|  | Behavioral skills |  |  |  | .56 | .93 | .02 |
|  | Intervention group | 12 | 26.58±6.42 | 35.33±7.33 |  |  |  |
|  | Control group | 8 | 27.63±6.84 | 30.13±6.90 |  |  |  |

**Table S3** Results of the generalized estimating equation model analysis

| Group | N | n（%） | | *P* | | |
| --- | --- | --- | --- | --- | --- | --- |
|  |  | Baseline | Postintervention | Interaction term | Group effect | Time effect |
| sleep duration |  | | | | | |
| ≤6h |  |  |  | <.001 | .93 | <.001 |
| Intervention group | 113 | 13（11.5） | 8（7.1） |  |  |  |
| Control group | 113 | 12（10.6） | 14（12.4） |  |  |  |
| 6.01~8.00h |  |  |  |  |  |  |
| Intervention group | 113 | 72（63.7） | 85（75.2） |  |  |  |
| Control group | 113 | 57（50.4） | 90（79.6） |  |  |  |
| >8h |  |  |  |  |  |  |
| Intervention group | 113 | 28（24.8） | 20（17.7） |  |  |  |
| Control group | 113 | 44（38.9） | 9（8.0） |  |  |  |
| sleep quality |  | | | | | |
| Preferably |  |  |  | .28 | .76 | .02 |
| Intervention group | 113 | 30（26.5） | 24（21.2） |  |  |  |
| Control group | 113 | 35（31.0） | 20（17.7） |  |  |  |
| Normal |  |  |  |  |  |  |
| Intervention group | 113 | 75（66.4） | 81（71.7） |  |  |  |
| Control group | 113 | 67（59.3） | 82（72.6） |  |  |  |
| Poor |  |  |  |  |  |  |
| Intervention group | 113 | 8（7.1） | 8（7.1） |  |  |  |
| Control group | 113 | 11（9.7） | 11（9.7） |  |  |  |
| somatic-psychological symptoms |  | | | | | |
| Dizziness (yes) |  |  |  | .39 | .93 | .68 |
| Intervention group | 113 | 23（20.4） | 21（18.6） |  |  |  |
| Control group | 113 | 20（17.7） | 25（22.1） |  |  |  |
| Eye discomfort (Yes) |  |  |  | .56 | .22 | .73 |
| Intervention group | 113 | 45（9.8） | 44（38.9） |  |  |  |
| Control group | 113 | 50（44.2） | 54（47.8） |  |  |  |
| Gastrointestinal problems (Yes) |  |  |  | .67 | .30 | .50 |
| Intervention group | 113 | 32（28.3） | 33（29.2） |  |  |  |
| Control group | 113 | 25（22.1） | 29（25.7） |  |  |  |
| Low mood (yes) |  |  |  | .91 | .18 | .18 |
| Intervention group | 113 | 28（24.8） | 33（29.2） |  |  |  |
| Control group | 113 | 21（18.6） | 26（23.0） |  |  |  |
| Anxiety (yes) |  |  |  | .65 | .20 | .47 |
| Intervention group | 113 | 38（33.6） | 33（29.2） |  |  |  |
| Control group | 113 | 29（25.7） | 28（24.8） |  |  |  |
| Difficulty concentrating (yes) |  |  |  | .21 | .13 | .08 |
| Intervention group | 113 | 37（32.7） | 39（34.5） |  |  |  |
| Control group | 113 | 24（21.2） | 35（31.0） |  |  |  |

**Table S4** Results of the subgroup analysis using generalized estimating equation model

|  | Group | N | n（%） | | *P* | | |
| --- | --- | --- | --- | --- | --- | --- | --- |
| Subgroup |  |  | Baseline | Postintervention | Interaction term | Group effect | Time effect |
| mild Internet addiction | sleep duration |  | | | | | |
|  | ≤6h |  |  |  | <.001 | .59 | <.001 |
|  | Intervention group | 101 | 12（11.9） | 8（7.9） |  |  |  |
|  | Control group | 105 | 10（9.5） | 12（11.4） |  |  |  |
|  | 6.01~8.00h |  |  |  |  |  |  |
|  | Intervention group | 101 | 65（64.4） | 75（74.3） |  |  |  |
|  | Control group | 105 | 52（49.5） | 85（81.0） |  |  |  |
|  | >8h |  |  |  |  |  |  |
|  | Intervention group | 101 | 24（23.8） | 18（17.8） |  |  |  |
|  | Control group | 105 | 43（41.0） | 8（7.6） |  |  |  |
|  | sleep quality |  | | | | | |
|  | Preferably |  |  |  | .33 | .61 | .006 |
|  | Intervention group | 101 | 29（28.7） | 21（20.8） |  |  |  |
|  | Control group | 105 | 33（31.4） | 19（18.1） |  |  |  |
|  | Normal |  |  |  |  |  |  |
|  | Intervention group | 101 | 66（65.3） | 75（74.3） |  |  |  |
|  | Control group | 105 | 63（60.0） | 76（72.4） |  |  |  |
|  | Poor |  |  |  |  |  |  |
|  | Intervention group | 101 | 6（5.9） | 5（5.0） |  |  |  |
|  | Control group | 105 | 9（8.6） | 10（9.5） |  |  |  |
| moderate to severe Internet addiction | sleep duration |  | | | | | |
|  | ≤6h |  |  |  | <.001 | .59 | <.001 |
|  | Intervention group | 12 | 1（8.3） | 0（0.0） |  |  |  |
|  | Control group | 8 | 2（25.0） | 2（25.0） |  |  |  |
|  | 6.01~8.00h |  |  |  |  |  |  |
|  | Intervention group | 12 | 7（58.3） | 10（83.3） |  |  |  |
|  | Control group | 8 | 4（50.0） | 5（62.5） |  |  |  |
|  | >8h |  |  |  |  |  |  |
|  | Intervention group | 12 | 4（33.3） | 2（16.7） |  |  |  |
|  | Control group | 8 | 2（25.0） | 1（12.6） |  |  |  |
|  | sleep quality |  | | | | | |
|  | Preferably |  |  |  | .33 | .61 | .006 |
|  | Intervention group | 12 | 1（8.3） | 3（25.0） |  |  |  |
|  | Control group | 8 | 33（31.4） | 1（12.5） |  |  |  |
|  | Normal |  |  |  |  |  |  |
|  | Intervention group | 12 | 9（75.0） | 6（50.0） |  |  |  |
|  | Control group | 8 | 63（60.0） | 6（75.0） |  |  |  |
|  | Poor |  |  |  |  |  |  |
|  | Intervention group | 12 | 2（16.7） | 3（25.0） |  |  |  |
|  | Control group | 8 | 9（8.6） | 1（12.5） |  |  |  |
